# Cadaveric study on feasibility of elbow joint denervation through a single posterior incision

**DOI:** 10.1016/j.xrrt.2026.100851

**Published:** 2026-08-17

**Authors:** Esmee Kwee, J. Michiel Zuidam, Jan Debeij, Roger van Riet, Joost Colaris, Denise Eygendaal, Caroline A. Hundepool

**Affiliations:** a Department of Plastic, Reconstructive Surgery and Handsurgery, Erasmus University Medical Centre, Rotterdam, The Netherlands; b Department of Plastic Surgery, Hand, Wrist and Elbow Unit, Haga Hospital, The Hague, The Netherlands; c Department of Orthopaedic Surgery, AZ Monica, Antwerp, Belgium; d Department of Orthopaedic Surgery, University Hospital Antwerp, Antwerp, Belgium; e Department of Orthopaedics and Sports Medicine, Erasmus University Medical Centre, Rotterdam, The Netherlands

**Keywords:** Elbow joint, Osteoarthritis, Denervation, Articular branches, Joint innervation, Cadaveric study

## Abstract

**Background:**

Treatment options for painful elbow osteoarthritis are limited. When a conservative therapy and débridement fail, total elbow arthroplasty remains the only salvage procedure. However, it is costly, highly invasive, and unsuitable for younger, active patients because of strict activity restrictions and limited prosthesis longevity. While denervation is a well-established intervention for hand and wrist osteoarthritis, its application in the elbow remains underexplored. This cadaveric study assesses the accessibility of elbow joint articular branches (ABs) through a single posterior incision.

**Methods:**

Ten fresh-frozen upper extremities were dissected via a standardized 20-cm posterior midline incision. Eight nerves were targeted: the medial antebrachial cutaneous nerve, ulnar, ulnar collateral, median, posterior antebrachial cutaneous nerve, radial, radial collateral, and musculocutaneous nerve. For each nerve, accessibility, number of ABs, and distance from the corresponding epicondyle were recorded.

**Results:**

All 8 nerves and their ABs were successfully accessed in all 10 specimens. Medial nerves include the medial antebrachial cutaneous nerve (mean 1.3 ABs; 4.8 cm from medial epicondyle), ulnar nerve (1.3 ABs; 1.6 cm), ulnar collateral nerve (7.4 cm), and median nerve (1.2 ABs; 2.0 cm). Lateral nerves include the posterior antebrachial cutaneous nerve (1.8 cm from lateral epicondyle), radial nerve (1.4 ABs; 6.4 cm), radial collateral nerve (7.0 cm), and musculocutaneous nerve (1.0 AB; 1.2 cm).

**Conclusion:**

A single-incision posterior approach provides consistent access to all key nerves innervating the elbow joint. These findings demonstrate the anatomic feasibility of elbow denervation, while preserving the option for a future reconstructive surgery through the same incision.

Elbow osteoarthritis represents a significant clinical challenge, typically presenting with pain, stiffness, and impaired function.^4^ Treatment options for elbow osteoarthritis are limited. While conservative management and early surgical interventions, such as arthroscopic or open débridement, may provide relief from end-motion impingement symptoms due to osteophyte formation, these approaches generally fail to address mid-motion pain resulting from joint space narrowing.^4^^,^^12^^,^^18^^,^^19^^,^^23^ In advanced cases of the disease, salvage procedures including elbow arthroplasty may be indicated. However, such procedures are highly invasive, associated with considerable risks of complications, require strict postoperative activity restrictions, and demonstrate an average prosthesis survival rate of 80% at 10 years.^15^^,^^19^^,^^20^ As a result, elbow arthroplasty is typically reserved for elderly, less-active patients. This leaves younger, more-active individuals and elderly patients who are unwilling or unable to undergo such invasive procedures with persistent, debilitating elbow osteoarthritis.

In other osteoarthritic joints, denervation surgery has emerged as a less-invasive alternative to arthroplasty or arthrodesis. During denervation, the sensory branches to the joint are selectively resected, aiming to reduce pain while preserving joint function.^1^^,^^13^^,^^14^ Denervation has demonstrated great outcomes in the treatment of osteoarthritis of the hand and wrist, with studies reporting an average pain reduction of 61% and a patient satisfaction rate of 83% following wrist denervation.^21^ Nonetheless, data on denervation of the elbow joint remain very limited, with only one clinical study published to date. In 1948, Bateman described a three-incision approach for elbow denervation in patients with osteoarthritis, reporting significant pain reduction and restored range of motion in 10 of 11 cases.^1^ Given the scarcity of effective treatments, denervation may represent a promising alternative for managing elbow osteoarthritis.

The feasibility of elbow denervation has historically been questioned because of the joint's complex innervation. However, recent anatomic research by Laumonerie et al has renewed interest by confirming the feasibility of a two-incision anterior approach in a cadaveric study.^13^^,^^14^ Building upon these encouraging findings, we propose investigating the feasibility of performing elbow denervation via a single-incision posterior approach. As total elbow arthroplasty is performed through the same posterior incision, using a single-incision approach for denervation may reduce the risk of complications, such as soft tissue necrosis or scarring, by minimizing the number of surgical incisions. This study extends current knowledge and further validates the concept of elbow denervation.

The purpose of this study is to assess the accessibility of articular branches (ABs) of the ulnar, ulnar collateral, radial, radial collateral, median, musculocutaneous, posterior antebrachial cutaneous (PABCN), and medial antebrachial cutaneous nerves (MABCN) through a single-incision posterior approach (Fig. 1). In addition, we aim to map the ABs of the targeted nerves from a single-incision posterior approach by quantifying their number and determining their location relative to the corresponding epicondyles. These findings aim to establish the anatomic feasibility of elbow denervation as a basis for future clinical studies in patients with severe elbow osteoarthritis.

**Figure 1:**
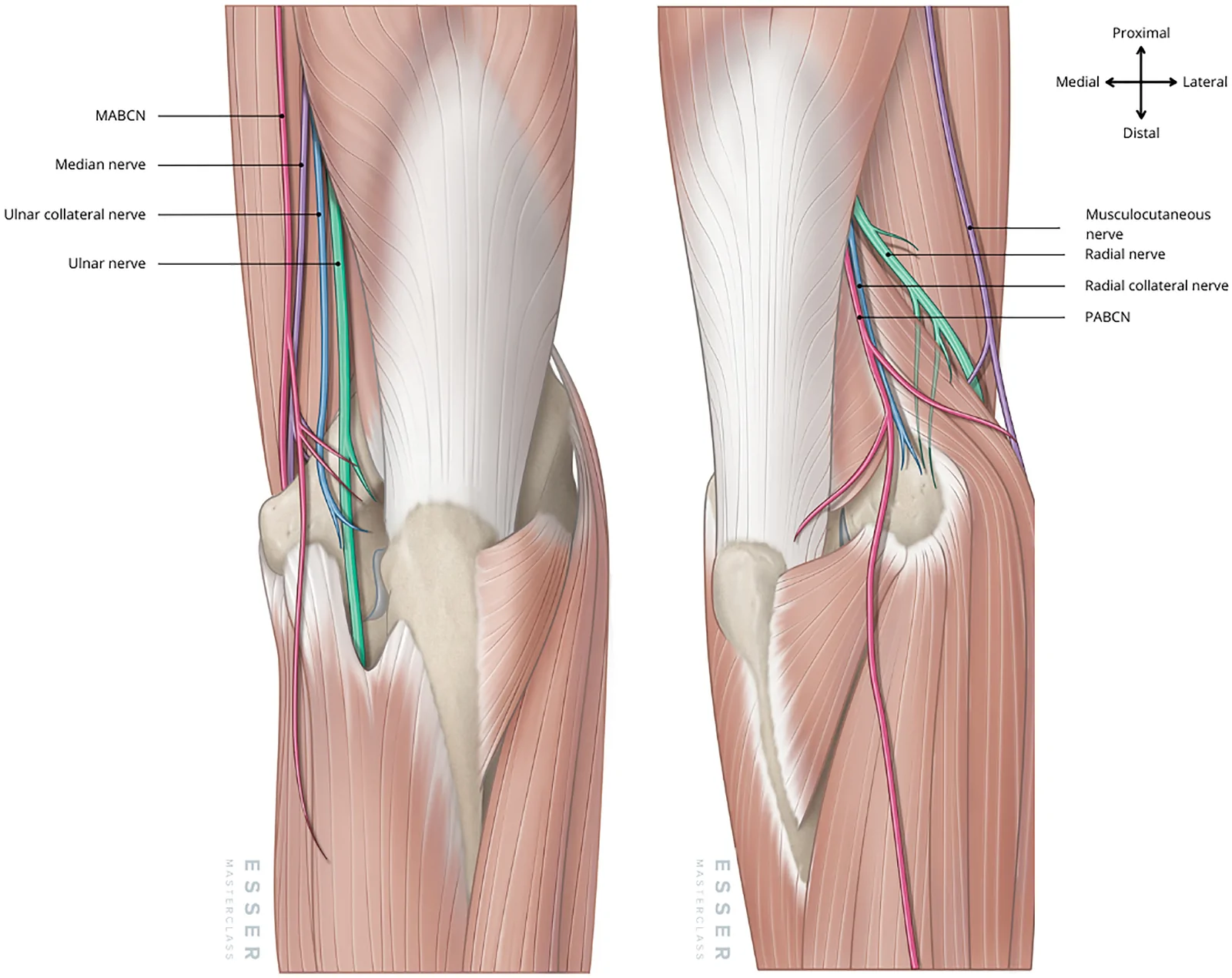
The articular branches of the nerves innervating the elbow joint from a posterior view. *MABCN*, medial antebrachial cutaneous nerve; *PABCN*, posterior antebrachial cutaneous nerve.

## Materials and methods

Ten fresh-frozen human cadaveric upper extremities were obtained from the Department of Anatomy of the Erasmus Medical Center as part of a national body donation program approved under Dutch law and regulations. All procedures involving human anatomic specimens were conducted in accordance with institutional and national ethical standards and followed the regulation for the use of donated bodies for scientific research and medical training.

In all specimens, a standardized posterior midline skin incision of approximately 20 cm was used. This incision length was selected to provide sufficient exposure while preserving the possibility of future reconstructive procedures, such as total elbow arthroplasty, through the same approach.

### Dissection protocol

All dissections were performed by a single experienced peripheral nerve surgeon to ensure consistency across specimens. Dissection was carried out systematically from medial to lateral. On the medial side, the MABCN, ulnar nerve, ulnar collateral nerve, and median nerve were identified around the medial epicondyle and distal humerus. The main trunk of each nerve was carefully isolated. On the lateral side, the PABCN, radial nerve, radial collateral nerve, and musculocutaneous nerve were identified in their respective anatomic intervals using the same posterior midline incision and lateral reflection of the skin flaps.

ABs were defined as branches with a clear trajectory toward the elbow joint capsule. For each targeted nerve, these branches were identified from their main trunk and traced to their attachment to the capsule and transected. Motor branches destined for muscle entry were left intact. In all specimens, the aim was to identify, follow, and resect every AB of the targeted nerves that could be accessed through the posterior midline approach.

### Measurements and data collection

For each specimen, the accessibility of every targeted nerve (present and identifiable through the posterior incision) was recorded. For each nerve, the total number of ABs identified was counted. Distances were measured from the point where each AB attached to the capsule to the corresponding medial or lateral epicondyle, using a digital caliper (perpendicular line to the epicondyle). The medial epicondyle was used as the reference point for branches from the MABCN, ulnar, ulnar collateral, and median nerves, and the lateral epicondyle for branches from the PABCN, radial, radial collateral, and musculocutaneous nerves. These bony landmarks were chosen because they are easily identifiable during a posterior midline approach. All data (presence of each nerve, number of ABs per nerve, and distances to the corresponding epicondyle) were recorded in a standardized data sheet for each specimen.

### Statistical analysis

Descriptive statistics were used throughout. Nerve accessibility was expressed as a count and percentage of specimens in which the nerve and its ABs could be identified. The number of ABs and their distance to the corresponding epicondyle were summarized as means with standard deviations and ranges.

### Surgical technique

#### Step 0: Preparation

Place the arm in a realistic position suitable for dissection.

#### Step 1: Skin incision

Make a straight posterior midline skin incision approximately 20 cm in length and create skin flaps.

#### Step 2: MABCN

Begin the dissection on the medial side. Identify the MABCN within the subcutaneous fat proximal to the medial epicondyle. Carefully isolate and resect the ABs to the elbow joint capsule while preserving cutaneous branches.

#### Step 3: Ulnar nerve

Locate the ulnar nerve posterior to the medial epicondyle. Perform a cubital tunnel release and neurolysis along the course of the ulnar nerve. Identify and resect all ABs encountered, preserving all motor branches.

#### Step 4: Ulnar collateral nerve

Locate the ulnar collateral nerve proximal to where it enters the medial intermuscular septum, parallel to the ulnar nerve, and perform neurolysis and resection.

#### Step 5: Median nerve

Identify the median nerve in the posterior compartment between the biceps and triceps muscles. Carefully resect all ABs, while preserving the muscular branches to the pronator teres and avoiding injury to the brachial artery.

#### Step 6: Ulnar nerve transposition

Transpose the ulnar nerve subcutaneously, especially in cases where future elbow arthroplasty is anticipated.

#### Step 7: Lateral dissection

Retract the skin laterally to provide access to lateral structures through the same midline incision.

#### Step 8: PABCN

Identify the PABCN in the subcutaneous fat proximal to the lateral epicondyle. Trace and resect its posterior branch supplying the elbow joint capsule.

#### Step 9: Radial nerve

Locate the interval between the triceps and brachialis muscles to identify the radial nerve. Perform neurolysis, preserving all muscular branches. Identify and resect all ABs encountered.

#### Step 10: Radial collateral nerve

Identify the radial collateral nerve, running with the lateral collateral vessels along the dorsal rim of the intermuscular septum, and resect the radial collateral nerve.

#### Step 11: Musculocutaneous nerve

Identify the interval between the biceps brachii and brachialis muscles to locate the musculocutaneous nerve. Isolate and resect all ABs encountered.

#### Step 12: Skin closure

Close the skin incision in a standard layered fashion.

## Results

All targeted nerves and their ABs were accessed through the posterior single-incision approach in all 10 fresh-frozen human cadaveric upper extremities. This approach successfully provided access to the MABCN, ulnar nerve, ulnar collateral nerve, median nerve, PABCN, radial nerve, radial collateral nerve, and musculocutaneous nerve (Fig. 1).

**Figure 2:**
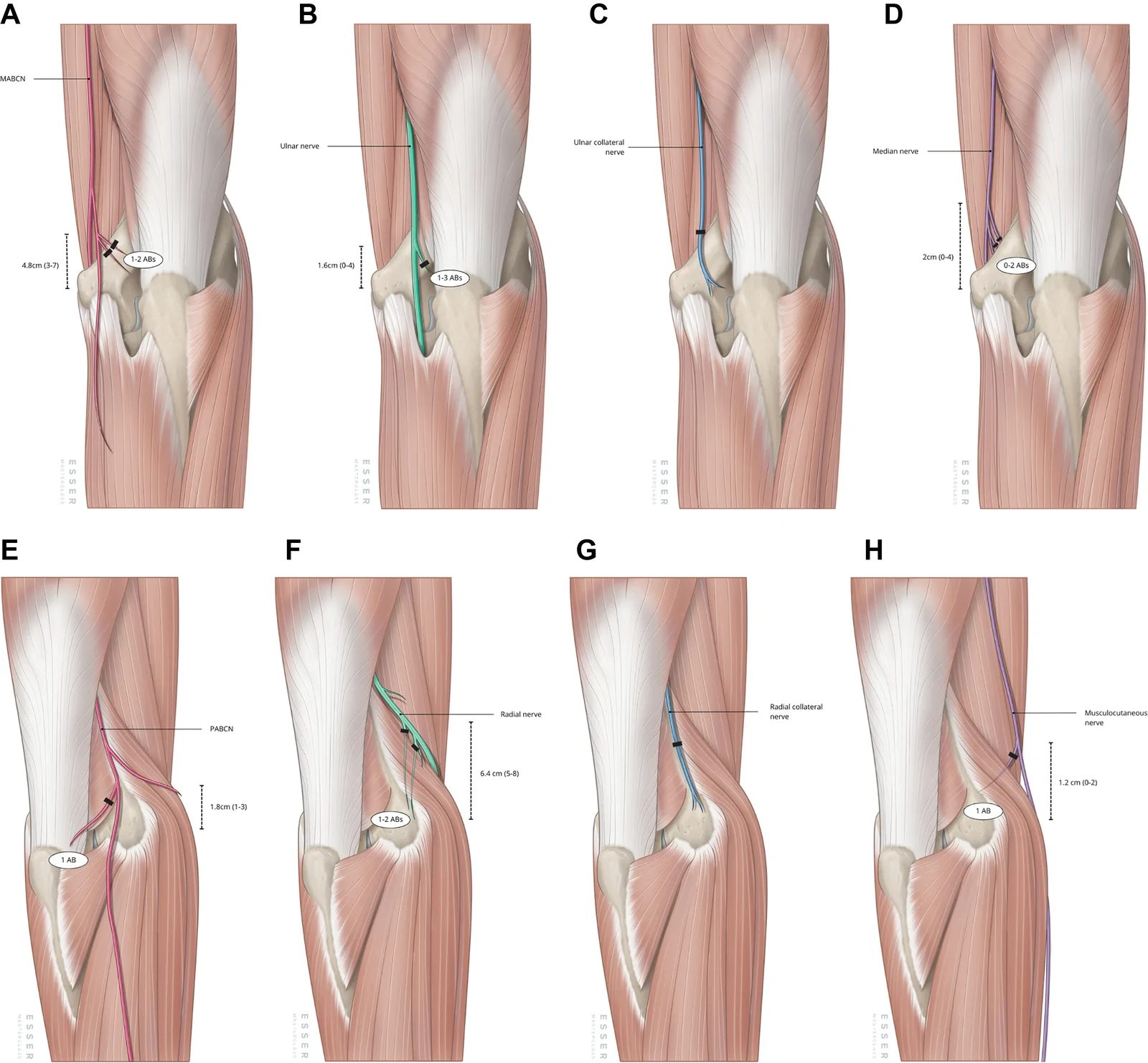
(**A**) ABs of the MABCN. (**B**) ABs of the ulnar nerve. (**C**) ABs of the ulnar collateral nerve. (**D**) ABs of the median nerve. (**E**) The PABCN is located laterally within the subcutaneous fat. It gives rise to a posterior AB that innervates the lateral capsule at a mean distance of 1.8 cm (range, 1-3 cm) from the lateral epicondyle. (**F**) ABs of the radial nerve. (**G**) ABs of the radial collateral nerve. (**H**) ABs of the musculocutaneous nerve. *MABCN*, medial antebrachial cutaneous nerve; *ABs*, articular branches; *PABCN*, posterior antebrachial cutaneous nerve.

The mean number of ABs and their distance from the corresponding epicondyle for each nerve are summarized in Table I, with schematic illustrations provided in Figure 2. On the medial side, the MABCN, ulnar nerve, and median nerve each gave off a mean of 1.2-1.3 ABs, whereas the ulnar collateral nerve was identified at a mean distance of 7.4 ± 0.7 cm from the medial epicondyle, after which it passes distally between the medial intermuscular septum (Fig. 2, *A*–*D*). On the lateral side, the radial nerve presented the highest mean number of ABs (1.4 ± 0.5), whereas the radial collateral nerve was identified at a mean distance of 7.0 ± 0.8 cm from the lateral epicondyle, after which it enters the intermuscular septum. The PABCN and musculocutaneous nerve were located closest to the lateral epicondyle, at mean distances of 1.8 ± 0.7 cm and 1.2 ± 0.8 cm, respectively (Fig. 2, *E*–*H*). Full per-specimen data are presented in Supplementary Table S1.

**Table I:** The number of articular branches identified and distances to corresponding epicondyle (cm) for each specimen.

Nerve	Reference epicondyle	Mean ABs ± SD (range)	Mean distance ± SD, cm (range)
MABCN	Medial	1.3 ± 0.5 (1-2)	4.8 ± 1.4 (3-7)
Ulnar nerve	Medial	1.3 ± 0.7 (1-3)	1.6 ± 1.0 (0-4)
Ulnar collateral nerve	Medial		7.4 ± 0.7 (6-8.5)
Median nerve	Medial	1.2 ± 0.6 (0-2)	2.0 ± 1.3 (0-4)
PABCN	Lateral		1.8 ± 0.7 (1-3)
Radial nerve	Lateral	1.4 ± 0.5 (1-2)	6.4 ± 0.8 (5-8)
Radial collateral nerve	Lateral		7.0 ± 0.8 (6-8)
Musculocutaneous nerve	Lateral	1.0 ± 0.0 (1)	1.2 ± 0.8 (0-2)

*ABs*, articular branches; *SD*, standard deviation; *MABCN*, medial antebrachial cutaneous nerve; *PABCN*, posterior antebrachial cutaneous nerve.

## Discussion

This cadaveric study demonstrates that a single-incision posterior approach provides consistent and reliable access to all ABs of the nerves innervating the elbow joint. The findings establish the feasibility of elbow denervation through a single posterior incision as a potential management option for elbow osteoarthritis, a condition for which current treatments remain limited, particularly in younger, more-active patients unsuitable for arthroplasty.^10^^,^^15^^,^^19^^,^^20^

In other joints, denervation has increasingly been adopted as a less-invasive alternative to, or as an initial step before, more extensive procedures such as arthroplasty or arthrodesis in patients with osteoarthritis.^1^^,^^13^^,^^14^^,^^21^ For the elbow, however, the main question has been whether denervation is surgically feasible given the extensive innervation of the elbow joint.

The posterior part of the medial capsule is predominantly innervated by the ulnar nerve, with additional contributions from the ulnar collateral nerve and the MABCN. ABs of the ulnar nerve are located posterior to the medial epicondyle, with consistent findings regarding their number and location reported in the literature.^1^, ^2^, ^3^^,^^8^^,^^9^^,^^14^^,^^16^^,^^17^ The ulnar collateral nerve, which originates from the radial nerve, runs parallel to the ulnar nerve and can be identified proximal to the medial intermuscular septum as it passes through the septum and then connects directly to the posteromedial capsule.^5^, ^6^, ^7^, ^8^^,^^14^ While the MABCN primarily supplies branches to the subcutaneous tissue, it also provides up to 2 ABs to the posterior part of the medial elbow capsule.^5^^,^^14^ The anterior part of the medial capsule receives innervation from the median and musculocutaneous nerve; however, there is variability in these contributions according to the literature. In this study, ABs from the median nerve originated either from the main trunk or from the motor branch of the pronator teres, while the musculocutaneous nerve consistently presented an AB near the lateral epicondyle.

The lateral capsule is primarily innervated by the radial nerve, with ABs originating proximally from the main radial trunk or muscular branches and distally from the posterior interosseous nerve.^1^, ^2^, ^3^^,^^5^^,^^7^^,^^8^^,^^11^^,^^14^^,^^17^^,^^22^ The current study includes the proximal ABs originating from the radial nerve or motor branches (brachioradialis), identified at a mean distance of 6.4 cm from the lateral epicondyle. The posterior part of the lateral capsule is also innervated by the radial collateral nerve and PABCN. The radial collateral nerve, coursing along the lateral intermuscular septum, attaches directly to the elbow joint capsule and was identified at a mean distance of 7 cm proximal to the lateral epicondyle.^11^^,^^14^ The posterior division of the PABCN was found at 1.8 cm from the lateral epicondyle. For the anterior part of the lateral capsule, the primary innervation comes from the radial nerve, with minor contributions from the musculocutaneous nerve.^3^^,^^5^

This study aimed to map the ABs of the targeted nerves by quantifying their number and identifying their location relative to the corresponding epicondyles. This approach was intended to facilitate a comparison with existing literature and determine if a posterior surgical approach would yield the same ABs. While our findings align partially with prior descriptions, variations exist in the literature. For example, De Kesel et al^5^ described all ABs innervating the elbow joint in detail, including those from muscular branches and nociceptive branches, whereas Laumonerie et al^14^ focused on nociceptive branches alone. This study includes ABs from nociceptive branches, as well as from any muscular branches with a clear trajectory to the elbow joint. Despite methodological differences across studies, we observed consistency within our cohort in the location of ABs relative to the epicondyles and the total number of ABs identified.

The consistent identification of ABs through a single posterior incision shows that elbow denervation could be a viable therapeutic option for pain reduction in patients with elbow osteoarthritis. However, the optimal number of ABs that should be targeted for effective elbow denervation remains unknown. To date, only one clinical study on elbow denervation has been reported. Bateman described a cohort of 11 patients with elbow osteoarthritis in the 1940s.^1^ After denervation of the ulnar, radial, median, and musculocutaneous nerves through 3 incisions, 10 of 11 patients experienced pain relief and restoration of painless movement, with no reported complications. Laumonerie et al suggested including denervation of the ulnar and radial collateral nerves, as these are both directly connected to the capsule, as well as denervation of the cutaneous nerves (MABCN and PABCN), which have also been shown to have ABs to the elbow joint.^14^ They demonstrated the feasibility of resecting the ABs of all these nerves through a two-incision anterior approach in their cadaveric study.

Although the two-incision anterior approach has been well described, we propose a single-incision posterior approach. This approach offers several potential advantages, including minimization of skin complications, necrosis, and scarring by reducing the number of incisions, as well as facilitation of future reconstructive procedures through the same incision. When performing this procedure clinically, it is recommended to use a nerve stimulator to differentiate between motor branches and ABs, ensuring precise identification and preservation of motor function. In addition, a subcutaneous transposition of the ulnar nerve can be performed if the nerve is found to be unstable following the neurolysis.

The main advantages of denervation over arthroplasty are that it eliminates pain while preserving joint function without imposing lifting restrictions, allows for shorter recovery time, is less costly, is not dependent on prosthesis longevity, and does not preclude future reconstructive procedures. Given these benefits, denervation is increasingly used for the management of chronic hand and wrist pain. A recent systematic review evaluated outcomes of denervation in the wrist, thumb, and fingers.^21^ Wrist denervation resulted in a mean reduction of 3.3 points on the visual analog scale for pain and a patient satisfaction rate of 83%. Denervation of the first carpometacarpal joint demonstrated a mean visual analog scale pain reduction of 5.4 points with 82% patient satisfaction. For finger denervation, pain decreased by 96% in the metacarpophalangeal joint, 81% in the proximal interphalangeal joint, and 100% in the distal interphalangeal joint. These outcomes are highly encouraging. Should elbow denervation prove similarly effective, it could offer substantial relief to patients suffering from chronic elbow pain for whom currently no suitable treatment options exist.

This study has several limitations. ABs were traced until a clear connection with the joint capsule was observed, allowing differentiation from muscular branches. However, the distinction between articular and muscular branches may remain uncertain without the use of intraoperative nerve stimulation. As an anatomic study, it did not assess clinical outcomes or evaluate potential complications associated with the larger subcutaneous dissection and more extensive skin flaps required for a single-incision posterior approach. It is important to emphasize that the clinical evidence for elbow denervation remains limited to one study. The absence of additional clinical trials underscores the critical need for further research in this area.

## Conclusions

This cadaveric study demonstrates the feasibility of identifying the key nerves responsible for elbow joint innervation and accessing its ABs through a single-incision posterior approach. The consistent identification of these branches suggests that this technique may offer a viable and less-invasive alternative to joint replacement.
