# Supplementary material for: Cadaveric study on feasibility of elbow joint denervation through a single posterior incision
Source: JSES Rev Rep Tech. 2026 Aug 17;6(4):100851. doi: 10.1016/j.xrrt.2026.100851 (PMC13594733; doi:10.1016/j.xrrt.2026.100851)
Supplement: Supplementary file 1 [file mmc1.docx]

**Supplementary Table 1.** Full per-specimen data

|  | 1 | 2 | 3 | 4 | 5 | 6 | 7 | 8 | 9 | 10 | Mean |
| --- | --- | --- | --- | --- | --- | --- | --- | --- | --- | --- | --- |
| MABCN | 2AB  5cm  5cm | 1AB  3cm | 2AB  6cm  6cm | 1AB  4cm | 1AB  3cm | 1AB  7cm | 1AB  3cm | 1AB  3cm | 1AB  5cm | 2AB  6cm  6cm | 1.3AB  4.8cm |
| Ulnar nerve | 3AB  2.5cm  1.5cm  0cm | 1AB  2cm | 1AB  1cm | 1AB  1cm | 1AB  1cm | 2AB  2.5cm  1cm | 1AB  1cm | 1AB  4cm | 1AB  1cm | 1AB  2cm | 1.3AB  1.6cm |
| Ulnar collateral nerve | 7cm | 8cm | 7cm | 7cm | 8cm | 7cm | 6cm | 8.5cm | 7cm | 8cm | 7.4cm |
| Median nerve | 2AB  2cm  1cm | 1AB  0cm | 2AB  4cm  2cm | 1AB  3cm | 2AB  4cm  1cm | 0AB | 1AB  3cm | 1AB  0cm | 1AB  2cm | 1AB  2cm | 1.2AB  2cm |
| PABCN | 1cm | 2cm | 1cm | 1cm | 1cm | 2.5cm | 2cm | 3cm | 2cm | 2cm | 1.8cm |
| Radial nerve | 2AB  7cm  6cm | 2AB  8cm  7cm | 2AB  7cm  5cm | 1AB  6cm | 2AB  7cm  6cm | 1AB  6cm | 1AB  6cm | 1AB  6cm | 1AB  5cm | 1AB  7cm | 1.4AB  6.4cm |
| Radial collateral nerve | 7cm | 7cm | 6cm | 6cm | 8cm | 6cm | 7cm | 8cm | 8cm | 7cm | 7cm |
| Musculocutaneous nerve | 1AB  0cm | 1AB  0cm | 1AB  2cm | 1AB  1cm | 1AB  2cm | 1AB  1cm | 1AB  2cm | 1AB  1cm | 1AB  1cm | 1AB  2cm | 1AB  1.2cm |

MABCN, medial antebrachial cutaneous nerve; PABCN, posterior antebrachial cutaneous nerve
