# Supplementary material for: Cadaveric study on feasibility of elbow joint denervation through a single posterior incision
Source: JSES Rev Rep Tech. 2026 Aug 17;6(4):100851. doi: 10.1016/j.xrrt.2026.100851 (PMC13594733; doi:10.1016/j.xrrt.2026.100851)
Supplement: Supplementary file 2 [file mmc2.docx]

**SUPPORTING FILE LEGENDS**

# Figures

**Figure 1.** The articular branches (ABs) of the nerves innervating the elbow joint from a posterior view. **Figure 2A.** The medial antebrachial cutaneous nerve (MABCN) is located within the subcutaneous fat. Its posterior branch gives rise to 1 or 2 ABs (mean 1.3), which innervate the posterior capsule at a mean distance of 4.8 cm (range, 3–7 cm) from the medial epicondyle. When 2 ABs are present, both originate from the same point.

**Figure 2B.** The ulnar nerve is located posterior to the medial epicondyle and supplies the posterior capsule with 1 to 3 ABs (mean 1.3) that originate at a mean distance of 1.6 cm (range, 0–4 cm) from the medial epicondyle. These branches arise at different levels but are all located within 0-4cm behind the medial epicondyle.

**Figure 2C.** The ulnar collateral nerve is identified running parallel to the ulnar nerve at a mean distance of 7.4 cm (range, 6-8.5 cm) proximal to the medial epicondyle. At this level, it is located proximal the entry of the medial intermuscular septum. Because it maintains a direct connection to the capsule, the ulnar collateral nerve can be transected without functional consequences other than denervation of the capsule.

**Figure 2D.** The median nerve is located between biceps and triceps muscles. It gives rise to a mean of 1.2 ABs (range, 0-2) at a mean distance of 2 cm (range, 0-4cm) from the medial epicondyle. The AB may originate directly from the trunk of the median nerve or from the motor branch of the pronator teres.

**Figure 2E.** The posterior antebrachial cutaneous nerve (PABCN) is located laterally within the subcutaneous fat. It gives rise to a posterior AB that innervates the lateral capsule at a mean distance of 1.8 cm (range, 1-3 cm) from the lateral epicondyle.

**Figure 2F.** The radial nerve is located between the triceps and brachialis muscles. It gives rise to a mean of 1.4 ABs (range, 1-2) at a mean distance of 6.4 cm (range, 5-8 cm) from the lateral epicondyle. The AB may originate directly from the trunk of the radial nerve or from the motor branch of the brachioradialis.

**Figure 2G.** The radial collateral nerve is identified parallel to the radial nerve at a mean distance of 7 cm (range, 6-8 cm) from the lateral epicondyle, after which it enters the lateral intermuscular septum. The radial collateral nerve is directly connected to the lateral capsule and can therefore be resected. **Figure 2H.** The musculocutaneous nerve runs between biceps brachii and brachialis muscles and provides 1 AB, at a mean distance of 1.2 cm (range 0-2cm) from the lateral epicondyle.
